# The Nexus of Training Duration, Body Image, Nutritional Practices, and Mental Health: Insights from a Strength Training Cohort

**DOI:** 10.3390/bs14040267

**Published:** 2024-03-23

**Authors:** Jorge Jiménez-Morcillo, Stephanie Rodriguez-Besteiro, Vicente Javier Clemente-Suárez

**Affiliations:** 1 Faculty of Sports Sciences, Universidad Europea de Madrid, Tajo Street, s/n, 28670 Madrid, Spain; jorge.jimenez2@universidadeuropea.es (J.J.-M.); vctxente@yahoo.es (V.J.C.-S.); 2 Grupo de Investigación en Cultura, Educación y Sociedad, Universidad de la Costa, Barranquilla 080002, Colombia

**Keywords:** strength training, body image, nutritional habits, psychological health, training frequency, wellness outcomes

## Abstract

This study investigated the intricate relationship between strength training and its effects on body image, psychological health, and nutritional habits. By examining 605 participants, divided into two groups based on training frequency, the research aimed to discern how varying intensities of training influenced different wellness facets. The investigation employed a comprehensive survey, gathering demographic data, training specifics, dietary patterns, and psychological characteristics, utilizing statistical tools for analysis. Results unveiled significant differences in dietary habits and psychological profiles between groups with higher and lower training frequencies. The group with more frequent training displayed less favourable health outcomes and suboptimal dietary habits, challenging the prevailing notion that increased training frequency leads to better health. The study emphasized the necessity of a balanced approach to physical training, highlighting the need for personalized strategies that encompass both physical and mental health considerations. The findings exposed the complexities of training regimens and their broader implications on individual health, suggesting that enhanced training frequency alone does not assure improved health outcomes. This research significantly contributed to the domain by providing insights into how the frequency of strength training could differentially affect health and well-being, offering valuable guidelines for fitness professionals and healthcare providers.

## 1. Introduction

The contemporary landscape has witnessed a substantial surge in the utilization of fitness facilities and participation in physical exercises, primarily motivated by aesthetic and health considerations [1]. Regular physical exercise is increasingly recognized for its role in mitigating the risk of chronic conditions such as type 2 diabetes, cardiovascular diseases, and certain forms of cancer [2]. Concurrently, the quest for physical fitness has ascended to cultural prominence, fueled in part by the rising influence of social media and fitness icons [3]. This preoccupation with physical aesthetics is not merely a societal trend but also harbors potential health implications, where the pursuit of aesthetic ideals and dissatisfaction with one’s physical appearance can lead to significant psychological and physical health consequences [4].

The constructs of body image and self-perception are profoundly influenced by cultural norms and personal experiences. Negative body image, defined by a distorted and often dissatisfactory view of one’s physique, has been linked to a spectrum of adverse effects, including low self-esteem, depression, and disordered nutritional practices [5]. These associations have been observed in women with notable distinctions in their manifestation and impact. Particularly, research indicates that females across various age demographics, especially in the adolescent to young adult age range (ages 15–30) [4], experience these negative consequences. However, the specific patterns and severity of these effects can differ significantly, suggesting the need for nuanced understanding and approaches in addressing body image concerns within these diverse groups [6]. Particularly, conditions like anorexia nervosa and bulimia nervosa often correlate with a distorted perception of body image, prompting extreme weight loss measures [7]. Additionally, studies have suggested that a poor body image is a precursor to disordered eating, especially prevalent among young women [8].

Building upon the analysis initiated in our prior research [9], where gender differences in body satisfaction perception and their correlation with nutritional habits, psychological traits, and physical activity among a strength training population were examined, we aim to further elucidate this intricate interplay [9]. Published in *Nutrients*, our preceding study laid the groundwork by shedding light on how interconnected factors impact body image across genders within this specific group. The current manuscript extends the scope of investigation, incorporating additional variables to not only validate and broaden the understanding of these themes but also to explore new dimensions related to body image and exercise. This approach addresses the previously identified limitations, adopting methodological enhancements to provide a more detailed and nuanced perspective, significantly contributing to the existing literature on the interrelation between body image perception, nutrition, psychology, and physical activity in strength training contexts. Physical exercise is a structured form of physical activity that is planned, repetitive, and purposefully aimed at improving or maintaining one or more components of physical fitness [10]. A positive body image tends to encourage engagement in physical exercises and the maintenance of an active lifestyle [11]. Conversely, regular physical exercise has been observed to positively impact body image and self-esteem, particularly for those focused on weight management [12]. However, body dissatisfaction may act as a barrier to participating in physical exercises, especially in social settings [13]. Hence, addressing body image concerns could be instrumental in promoting physical exercise, leading to improved health outcomes [14].

In addition to these core areas, the study also considered the societal and psychological factors that might influence an individual’s engagement in physical exercise. The role of socio-economic status, access to fitness resources, and prevailing societal norms regarding physical appearance are critical in shaping one’s approach to physical exercise [15]. Research indicated that disparities in access to fitness facilities and resources could lead to significant differences in physical exercise levels across different socio-economic groups, thereby influencing wellness outcomes [16].

Furthermore, the psychological impact of physical exercise extends beyond body image and wellness. The endorphin release associated with exercise is known to have mood-enhancing effects, potentially offering therapeutic benefits for individuals dealing with stress, anxiety, and depressive symptoms [17,18]. This aspect of physical exercise underscores its role not only as a tool for physical transformation but also as a catalyst for psychological resilience and emotional well-being [19]. By considering these broader implications, the current study provides a more nuanced understanding of the interconnections between physical exercise, mental health, and overall wellness.

This study delves into the intricate relationship between physical exercise, with a specific focus on training duration and its impact on body image and related wellness aspects. The main objective of this study is to explore the variations in body image perception, mental health, dietary practices, and training routines among participants involved in physical training programs of varying durations. We hypothesize that the intricacies of exercise duration exert a multifaceted influence on individual wellness, as delineated by three specific avenues. Initially, we posit that an increased training duration is positively correlated with enhanced body image perceptions [20], suggesting that prolonged engagement in physical activities fosters a more affirmative self-assessment and appreciation of bodily aesthetics [21]. Concurrently, we explore the premise that extended periods of physical training contribute to a broad spectrum of psychological health benefits, including the alleviation of stress, anxiety, and depressive symptoms, thus promoting a state of mental well-being integral to the holistic concept of wellness [22]. Lastly, our hypothesis extends to the assertion that individuals dedicated to longer training durations are more inclined towards adopting superior nutritional practices and adhering to disciplined training regimens, reflecting a comprehensive approach to wellness that integrates both physical and nutritional dimensions [23]. While previous studies have established a link between the duration of physical exercise and mental health outcomes, this research seeks to delve deeper into how these factors specifically influence body image and their interaction with other wellness-related variables.

## 2. Methods

### 2.1. Design

In the structuring of our study’s sample, participants were divided into two distinct groups based on their weekly training frequency, employing the 50th percentile as a critical dividing line. To calculate the sample size for our study, we employed Cohen’s d to estimate the effect size. This decision was based on the anticipated differences between groups in key variables of interest, such as body satisfaction, psychological health, and nutritional habits. Cohen’s d was determined by considering the expected difference between means of the groups, divided by the pooled standard deviation. This methodological choice allowed us to estimate the minimum number of participants required to detect a statistically significant difference with a power of 0.80 and an alpha level of 0.05. The calculation of Cohen’s d and subsequent sample size estimation were critical steps in ensuring that our study was adequately powered to test our hypotheses, thus enhancing the reliability and validity of our findings. The categorization criterion was centered around the median frequency of training sessions per week, as determined by the 50th percentile of our sample. Participants whose training frequency fell at or below the 50th percentile were classified into the lower training group (LTG). This group encapsulated individuals whose training frequency was less frequent, reflecting the lower half of the distribution in our sample. The LTG provided valuable insights into the effects of a less frequent exercise regimen, which is crucial for understanding the broader range of training habits in the population. Conversely, participants whose training frequency was above the 50th percentile were placed in the higher training group (HTG). This group represented a more active segment of our sample, engaging in training sessions more frequently than their LTG counterparts. The HTG allowed for a concentrated examination of the outcomes tied to a higher frequency and more consistent training schedule. This percentile-based division enabled a nuanced comparison between the two groups. It offered a more refined analysis by using a median split approach, which ensures a balanced comparison between the lower and higher frequency training participants.

### 2.2. Participants

In this investigation, we interviewed 590 participants engaged in strength training through an online survey, comprising 315 males and 275 females. For the calculation of the sample size, the SAS software v9.4. was utilized. The initial sample size was set at 620 subjects. Throughout the process, a dropout of 30 participants was observed, comprising 20 men and 10 women, resulting in a dropout rate of 4.84%. To enhance the reliability of the self-reported data, detailed instructions and examples on how to estimate their 1RM were provided, based on their current training loads. Participants accessed the survey either via a unique Google Forms link or by scanning a QR code. They were fully briefed on the study’s aims and methods before participating. Eligibility criteria demanded that both male and female participants be aged 26 to 43 years and have consistently participated in strength training 2 to 7 days per week for at least 12 months. The questionnaire’s reliability and validation were carefully considered; for newly developed questions, a pilot test was conducted to assess clarity and reliability. For dietary measures, visual aids and common household equivalents were provided to assist participants in accurately estimating food quantities. The specific criteria for the training regimen followed by the subjects of the study required that at least four of the total weekly sessions be specifically strength-based and at least two of these sessions be of an aerobic nature. In addition, all participants completed a Physical Activity Readiness Questionnaire (PAR-Q), and one of the exclusion criteria for the study was an affirmative response to two or more questions. This procedure ensured that none of the subjects presented significant health issues [24]. To further ensure response reliability, cross-validation checks were applied alongside control questions within the survey to identify and correct inconsistencies in self-reporting. Each participant’s response was scrutinized for accuracy and thoroughness to ensure response reliability. An initial analysis was conducted to spot any biases or unusual patterns in the responses. Participation was purely voluntary, with the option to withdraw at any time without repercussions. Each participant gave digital consent through a signed informed consent form, indicating their comprehension and agreement to partake in the study. The research conformed to the Helsinki Declaration’s principles (revised in Brazil, 2013) concerning ethical human research. Ethical approval was granted by the University Ethics Committee (CIPI/21/082), ensuring adherence to ethical standards.

### 2.3. Setting

In enhancing the rigor of our research design as outlined in Section 2.2, a key step involved instituting the ‘Participant Response Accuracy Commitment’ (PRAC). This mandatory agreement, signed by all study participants, was crucial in emphasizing the importance of accuracy in participant responses. By digitally endorsing the PRAC, participants committed to providing truthful and precise answers, a pledge that was vital to the integrity and validity of our study’s outcomes. This novel method aimed not only to reduce potential biases in the data but also to underscore our dedication to conducting research that is ethically sound, adhering to the principles of the Helsinki Declaration (revised in Brazil, 2013).

**Demographic and body measurements**: Gender, age, height, weight, and body mass index (BMI) were recorded.

**Resistance training specifics:** Participants reported their weekly training sessions, including both aerobic and strength exercises. Aerobic training was quantified in weekly minutes, while strength training intensity was gauged based on weekly training percentages below 50%, between 50% and 70%, between 70% and 85%, and above 85% of their one-repetition maximum (1RM). Back squat, deadlift, and bench press 1RM percentages were also noted. In our methodology, the 1RM (one-repetition maximum) was not directly measured through physical testing due to the constraints of conducting an online survey. Instead, participants were asked to report their estimated 1RM based on their most recent training sessions, employing widely recognized estimation techniques described in strength and conditioning research [25]. To facilitate accurate reporting, we provided detailed instructions and examples within the Google Forms survey on how to estimate their 1RM based on their current training loads and the maximum number of repetitions they could perform at a given weight.

**Dietary patterns:** We utilized a food consumption frequency questionnaire to assess the intake of various items like juices [250 mL], water [250 mL glasses], alcoholic (250 mL) and fermented beverages (0250 mL), soft drinks (250 mL), energy drinks (250 mL), milk (250 mL glasses), fruits (90 g), bakery/sweets (90 g), meat (150 g), fish (150 g), legumes (200 g), pasta or rice (150 g), vegetables (200 g), bread (50 g), fast food (180 g), whole foods (150 g), gel (40 g), muesli bars (150 g), and protein drinks (300 mL). This was based on previous research [26,27].

**Nutritional habits:** Data on the frequency of takeout meals, dining out, and home-cooked meals were gathered. These data were collected using the diet quality index (DQI) [28].

**The Big Five Inventory:** Employed in our study’s methodology to measure participants’ personality traits, this inventory, with a reliability alpha coefficient of 0.73, assesses core personality dimensions—openness, conscientiousness, extraversion, agreeableness, and neuroticism [29].

**The Spielberger State–Trait Anxiety Inventory (reduced Spanish adaptation):** Utilized in our study to evaluate anxiety levels among participants, this version of the inventory boasts a high reliability alpha coefficient of 0.93. It is designed to assess both the transient and enduring aspects of anxiety [30].

**The Acceptance and Action Questionnaire II (Spanish version):** In our study, this questionnaire was employed to assess experiential avoidance or psychological inflexibility among participants, featuring a reliability alpha coefficient of 0.84 [31].

**The UCLA Loneliness Scale (Spanish version):** This scale, with a reliability alpha coefficient of 0.94, was used in our research to measure loneliness among participants [32].

**The Zung Depression Scale (adapted Spanish version for the COVID-19 context):** This scale, demonstrating an alpha coefficient of 0.09 with high sensitivity and specificity over 80%, was utilized in our study to assess depression levels among participants [33].

**Body image satisfaction:** To distinguish body image satisfaction between strength-trained men and women, we measured different levels with various body parts using the nutritional disorder inventory [34], which included a numeric scale (1 (never) to 6 (always)) and a body silhouette scale (1 being thinnest, 9 most voluminous).

### 2.4. Data Analysis

Statistical analysis was performed using SPSS version 24.0. Descriptive statistics were calculated, and the normality and homogeneity of the data were assessed using Kolmogorov–Smirnov tests. An independent t-test analyzed differences in nutrition, sociodemographic, academic, and psychological factors, with the significance set at *p* ≤ 0.05. Additionally, to investigate the relationships implied by our hypotheses and discussed in our findings, multivariate analysis techniques, including Pearson correlation analysis and multiple linear regression, were applied. These analyses allowed us to explore the associations between specific variables, such as strength training habits, dietary patterns, and psychological dimensions. For instance, we examined the relationships between the frequency and intensity of strength training and body image satisfaction and anxiety levels, controlling for demographic factors and dietary habits. These statistical methods were chosen to unravel the complex interactions among the study variables, providing a deeper understanding of how various aspects of strength training and psychological health are interconnected within our sample. The selection of these analyses was predicated on the understanding that identifying differences is only the first step; comprehending the underlying dynamics that link training behavior with mental and physical health outcomes was crucial to the core of our hypotheses. Given our exploratory approach, the selection of these analyses was grounded in the premise that identifying differences represents merely the initial step; comprehending the underlying dynamics that link training behavior with mental and physical health outcomes is paramount to the core of our exploratory endeavor.

## 3. Results

In the analysis of the obtained results, significant disparities were observed in participant demographics between the two training groups. It was evident that participants in the higher training group exhibited a statistically significant lower mean age in comparison to those in the lower training group (Table 1).

In the nutritional data analysis, discernible differences in dietary habits were identified between the lower training group and the higher training group. Notable disparities were observed in the weekly consumption of various food items. The lower training group showed a distinct pattern in the consumption of items such as cups of alcohol, energy drinks, and milk glasses compared to the higher training group. Similarly, there were significant differences in the weekly intake of fermented dairy products, sweets and pastries, cheese, eggs, meat, and fish. The consumption patterns of legumes, pasta, fruits, both raw and cooked vegetables, bread, whole foods, fast food, protein drinks, gels, and muesli bars also varied markedly between the groups. These findings suggest divergent dietary preferences and consumption habits between the two groups, which may have implications for their overall nutritional status and health outcomes (Table 2).

In the psychological characteristics analysis, marked differences were identified between the lower training group and the higher training group. The analysis highlighted significant variations in traits such as extraversion, with one group exhibiting notably higher or lower levels compared to the other. Distinct contrasts were also observed in levels of pleasantness and scrupulousness. Further, the groups differed significantly in terms of neuroticism and openness to experience. Additionally, there were notable disparities in the Zung score, which is a measure of psychological well-being. Body satisfaction levels also varied considerably between the two groups. These results indicate distinct psychological profiles and well-being measures between the lower training group and the higher training group, suggesting the influence of training habits on psychological characteristics (Table 3).

In our training data analysis, significant distinctions emerged between the lower training group and the higher training group, elucidating a comprehensive spectrum of training frequencies, intensities, and health-related symptomologies. Notably, the higher training group engaged in a greater number of training sessions per week, dedicating more minutes to aerobic exercises, which underscored their elevated commitment to training volume. This group also exhibited higher bench press repetition maximum (RM) in kilograms, suggesting superior strength achievements. Health-wise, the lower training group reported a higher incidence of gastritis or heartburn, alongside increased occurrences of dry throat sensation and dental sensitivity, pointing towards the physiological cost of their training regimen. Additionally, the analysis unveiled significant variations in training intensity distribution: the lower training group allocated a larger percentage of their weekly training at sub-maximal loads (below fifty percent of maximum load), whereas the higher training group predominantly trained at higher intensities, specifically within the seventy to eighty-five percent range of maximum load and even more conspicuously above eighty-five percent. These insights reflect the divergent training methodologies and objectives pursued by the two groups, with the higher training group’s approach emphasizing both higher volume and intensity, aligning with their pronounced strength and endurance outcomes (Table 4).

## 4. Discussion

In the context of this investigation, it is imperative to reiterate the foundational objective as delineated in the introductory segment. The primary aim was to analyze how body image perception, psychological well-being, nutritional habits, and training patterns vary among individuals engaging in physical training programs of different lengths. This objective guides our methodical examination of the interplay between the duration of physical training and its consequent impact on a spectrum of wellness dimensions. While our study anticipated positive correlations between prolonged physical activity and aspects such as body image, psychological health, and nutritional practices, the findings were more complex. The higher training group, despite more frequent training, showed adverse health effects and less optimal nutritional habits, such as increased alcohol and energy drink consumption, contradicting the expected uniformly positive outcomes of extended physical exercise.

In our study, we observed significant differences in most of the analyzed variables. Initially, they were consistent with the existing literature [35]. First and foremost, a significant difference was observed between the training duration and weekly frequency, indicating that training sessions tend to be shorter as the frequency of training increases. Furthermore, in line with prior studies [36,37], athletes dedicating less time to physical activity are seen to engage in higher volumes of aerobic training. This could be attributed to the popularity of running among the general population [38] or the belief that cardiovascular exercise has a more substantial impact on weight loss compared to strength training, a notion fully addressed by scientific evidence [39]. According to the results obtained in our study, a lower training volume is associated with adaptations to strength training. Some research already suggests that to enhance performance in strength sports, it is not necessary to train at a higher volume [40]. The previous literature and studies confirm our results [41,42,43]; athletes who train at a lower intensity spend less time on their training sessions. This trend continues when the intensity is medium–high. However, the trend reverses when training intensity is higher; in this scenario, athletes dedicate more time to their training sessions. As per previous studies, this is because high-performance athletes require training demands of greater volume and intensity [44]. Our study also examined the effects of health and its relationship with training. The results were quite conclusive: longer training duration leads to more adverse health effects; specifically, the higher training group experienced increased dental sensitivity, more gastritis, and greater throat dryness. These findings align with what is established in the literature, suggesting that excessive training duration can lead to adverse health effects due to immunological depression [45].

Regarding the findings based on the nutrition section, there is a notable disparity in outcomes between the lower training group and the higher training group. Concerning alcohol consumption in relation to training duration, there is limited literature available [46], yet the existing studies [47,48] do not offer conclusive results. Our findings, however, are unequivocal: subjects engaging in lengthier training sessions consume more alcohol. Furthermore, with respect to the intake of energy drinks, our results align with the previous literature [49]; individuals dedicating more time to training consume more energy beverages. This could potentially be attributed to the high caffeine content of these drinks, often used as an ergogenic aid in athletes [50]. Paradoxically, regarding food consumption, our data indicate a higher intake of dairy and fermented dairy products, eggs, meat, fish, legumes, pasta, fruit, vegetables, and whole foods among subjects training for shorter durations compared to those undergoing longer training sessions. A previous research study suggests the opposite, indicating that an extended training duration necessitates increased caloric intake to meet athletes’ energy demands [51]. Moreover, a diet rich in protein sources such as fish, meat, eggs, legumes, and dairy is recommended for sports requiring long-duration training sessions [52], particularly in strength sports like powerlifting or weightlifting [53]. The literature is unequivocal regarding carbohydrate consumption: it is essential to meet the energy demands of athletic practice [54]. The findings of our study concerning nutritional habits should prompt reflection among the athletic population. Nonetheless, our results indicate a higher consumption of specific food groups in subjects whose training sessions are longer; these include fast food, whey protein drinks, gels, and muesli bars. There are studies corroborating our findings. The intake of high-calorie foods like fast food or muesli bars provides rapid energy [55] in strength sports [56], or for those with aesthetic objectives, they are used to increase muscle mass [57]. However, long-term consumption of these foods may have adverse effects on cardiovascular health [58]. Regarding the intake of whey protein beverages, our results are supported by previous studies; whey protein consumption is recommended in strength sports where training involves high volumes and durations [59].

The results obtained indicates that those people who belong to the group with higher training, presents more extroverted personality and tend to be more pleasant, with a greater openness to try new experiences and likewise greater traits of neuroticism and scrupulousness. There is considerable scientific evidence pointing to the benefits that physical exercise can have on the personality. Lu and Hu [60] noted that extraversion is significantly correlated with almost all types of sport and leisure practice; however, neuroticism would not be related in the same way [61]. In this line, significant results were also found in the relationship between higher levels of training and lower levels of depression. The scientific evidence indicates that physical exercise has great benefits on mental health, especially on anxiety and depression [62,63]. Borrega et al. indicate that high-intensity interval training and moderate-intensity training reduce stress, anxiety, and depression and increase resilience. As in our research, they also found lower scores on the depression scale in those participants who exercised at a higher intensity compared to those who exercised more moderately [64]. Other studies have found a relationship between training for at least 6 weeks and the reduction in depressive symptomatology [65]. In this line, a positive body image is related to lower psychological problems, such as depression and social avoidance [66]. Likewise, our results point out that the higher training group has better levels of body satisfaction. This is related to published research that reports that health-focused exercises are associated with positive body image and healthy nutritional habits [67]. In this line, our results are congruent with those found where it is shown that physical exercise improves appearance and physical perception. Likewise, in relation to body satisfaction, Añez et al. showed that physical activity had a greater effect on the female gender than on the male gender [68]. This influence of physical activity on appearance, self-esteem, and body dissatisfaction could be due to the role of physical exercise. Some longitudinal studies [69,70] have shown that moderate to vigorous physical activity changes the perception of self-concept through changes in body image and its effect on appearance and self-esteem [71]. In the context of our study on training duration and body image from a wellness perspective, it is relevant to acknowledge the findings of Sabiston et al. [72], who, in their scoping review on body image, physical activity, and sport, underscore the bidirectional influence between engagement in physical activities and body image perception. Their study highlights how physical activity can have both positive and negative impacts on body image, emphasizing the importance of promoting sports practices that nurture a healthy and positive view of the body. These findings support our research, suggesting that an appropriate and tailored training duration could be crucial in enhancing body image and therefore overall well-being [73,74,75,76]. Overall, it is relevant to acknowledge the findings of Lindheimer [77], who quantified the placebo effect in psychological outcomes of exercise training. Their meta-analysis reveals the complexity of measuring exercise effects, suggesting perceived benefits may not solely derive from physical aspects, which aligns with our observation of the nuanced impact of training duration on body satisfaction and well-being. This underscores the necessity of considering psychological dimensions in evaluating the outcomes of physical training. Although there is not much published literature related to these variables, our results indicate that the higher training group has higher levels of experiencing frequent gastritis or heartburn, frequent dry throat sensation and frequent dental sensitivity.

This study, while comprehensive in its approach, has certain limitations. The reliance on self-reported data via online surveys introduces potential biases, such as inaccurate self-assessment or response bias. The age range and specific focus on individuals engaged in strength training may limit the generalizability of the findings. Future research could expand the scope by including a wider age range and various forms of physical activity. Longitudinal studies would be beneficial to understand the dynamic interplay between physical training, psychological well-being, and dietary habits over time. Additionally, exploring the impact of different training intensities and durations across diverse socio-demographic backgrounds could provide a more holistic understanding of the subject. One notable limitation of our research is the lack of a control group, which would have significantly strengthened our analysis. Unfortunately, due to limitations in the sample size and the timing of participant access, it was not possible to include a control group in this study.

The findings of this study offer valuable practical applications for fitness industry professionals and healthcare providers. They highlight the necessity of developing personalized training and nutrition plans, especially for individuals engaged in strength training, to optimize physical and psychological health outcomes. This research underscores the importance of incorporating comprehensive wellness strategies that address both physical training and mental health considerations. For future implementation, these insights advocate for the integration of mental health screening and dietary counselling as part of fitness programs. Furthermore, the study accentuates the need for more inclusive and diverse fitness resources across different socio-economic groups, promoting holistic health approaches within community health initiatives and public health policies.

## 5. Conclusions

The study reveals a nuanced relationship between strength training, body image, psychological well-being, and nutritional habits. Notably, increased training frequency was associated with less favorable health outcomes, including poorer nutritional choices and heightened psychological traits like neuroticism, challenging traditional views on physical training benefits. These findings suggest the importance of a balanced, holistic approach to strength training that integrates both physical and psychological health considerations. Moreover, they highlight the need for personalized training and nutrition strategies, underscoring the role of socio-economic factors in access to fitness resources and the influence of societal norms on body image. Future research should focus on a more diverse demographic and longitudinal studies to further explore these relationships. This study contributes significantly to understanding the complex dynamics between physical training and overall wellness, offering practical insights for fitness professionals and healthcare providers in developing comprehensive wellness programs.

## Figures and Tables

**Table 1:** Comparison of demographic, educational, and employment variables related to training duration and body satisfaction from a wellness perspective.

					95% Confidence Interval of the Difference		
Variable	Lower Training Group	Higher Training Group	T	P	Lower	Upper	Cohen’s D
Age (Years)	20.1 ± 9.2	24.2 ± 6.8	3.111	0.002	0.849	3.759	0.34
Height (cm)	154.3 ± 9.1	172.7 ± 18.8	0.425	0.671	−2.036	3.162	0.03
Weight [kg]	72.0 ± 11.3	70.4 ± 11.6	1.542	0.123	−0.417	3.470	0.12
Body Mass Index (kg/m^2^)	19.7 ± 2.0	31.2 ± 127.6	−0.736	0.462	−26.523	12.066	−0.07

**Table 2:** Analysis of nutritional data and its impact on body satisfaction and training duration from a wellness perspective.

					95% Confidence Interval of the Difference		
Variable	Lower Training Group	Higher Training Group	T	P	Lower	Upper	Cohen’s D
**Days nutritional out of home**	1.5 ± 1.5	1.4 ± 1.3	0.849	0.396	−0.127	0.322	0.071
**Days ordering takeout**	0.6 ± 0.8	0.6 ± 0.8	0.362	0.718	−0.112	0.162	0.0
**Cook most days (1–5 scale)**	1.7 ± 0.9	1.7 ± 1.0	−0.549	0.583	−0.200	0.113	0.0
**Satisfaction with weigh (1–5 scale)**	2.0 ± 0.7	2.1 ± 0.7	−0.659	0.510	−0.169	0.084	−0.143
**Daily water glasses**	3.5 ± 1.8	3.7 ± 1.8	−1.569	0.117	−0.523	0.058	−0.111
**Fruit juice consumption [mL] [weekly]**	2.4 ± 0.9	2.3 ± 0.9	0.268	0.789	−0.134	0.176	0.111
**Glass of alcohol consumption [mL] [weekly]**	2.6 ± 0.7	2.5 ± 0.7	0.768	0.443	−0.070	0.160	0.143
**Beer consumption [mL] [weekly]**	2.4 ± 0.8	2.0 ± 1.3	0.495	0.620	−0.104	0.171	0.371
**Cups of alcohol consumption [mL] [weekly]**	2.0 ± 1.3	2.7 ± 0.6	−7.856	0.002	−0.841	−0.504	−0.691
**Cola/soda consumption [mL] [weekly]**	2.2 ± 1.4	2.4 ± 0.9	−1.724	0.085	−0.356	0.023	−0.170
**Energy drink consumption [mL] [weekly]**	2.0 ± 1.4	2.6 ± 0.7	−6.802	0.003	−0.820	−0.453	−0.542
**Milk glasses consumption [mL] [weekly]**	3.4 ± 2.7	2.6 ± 1.5	4.664	0.004	0.490	1.200	0.366
**Fermented dairy consumption [g] [weekly]**	3.0 ± 2.3	2.4 ± 1.4	3.583	0.001	0.253	0.868	0.315
**Sweets/pastry consumption [g] [weekly]**	2.1 ± 1.4	2.3 ± 0.8	−1.817	0.070	−0.375	0.014	−0.175
**Cheese consumption [g] [weekly]**	2.6 ± 1.9	2.2 ± 1.2	2.977	0.003	0.137	0.670	0.252
**Eggs consumption [g] [weekly]**	3.2 ± 2.8	2.4 ± 1.5	4.560	0.003	0.485	1.220	0.356
**Meat consumption [g] [weekly]**	3.4 ± 2.7	2.3 ± 1.6	5.591	0.002	0.668	1.393	0.496
**Fish consumption [g] [weekly]**	2.8 ± 2.0	2.0 ± 1.1	5.221	0.001	0.444	0.987	0.496
**Processed meat consumption [g] [weekly]**	2.5 ± 1.8	2.4 ± 1.2	0.774	0.439	−0.155	0.356	0.065
**Legume consumption [g] [weekly]**	2.4 ± 1.6	2.1 ± 1.1	2.356	0.019	0.044	0.492	0.219
**Rice consumption [g] [weekly]**	2.9 ± 2.5	2.3 ± 1.3	3.428	0.24362	0.897	0.744	0.301
**Weekly pasta [g] consumption**	2.7 ± 2.2	2.3 ± 1.3	2.966	0.003	0.152	0.750	0.221
**Weekly fruit consumption [g]**	3.9 ± 3.3	2.7 ± 1.7	5.427	0.001	0.749	1.599	0.457
**Weekly raw vegetable [g] consumption**	3.4 ± 2.9	2.3 ± 1.4	5.673	0.002	0.695	1.432	0.483
**Weekly cooked vegetable [g] consumption**	3.4 ± 2.9	2.4 ± 1.4	5.327	0.001	0.639	1.386	0.439
**Weekly bread [g] consumption**	3.0 ± 2.6	2.6 ± 1.4	2.564	0.011	0.103	0.781	0.192
**Weekly whole food [g] consumption**	3.0 ± 2.6	2.5 ± 1.4	3.092	0.002	0.194	0.869	0.239
**Weekly fast food [g] consumption**	2.0 ± 1.2	2.3 ± 0.7	−4.070	0.001	−0.488	−0.170	−0.305
**Weekly protein drink [mL] consumption**	2.3 ± 1.9	2.6 ± 0.9	−2.450	0.015	−0.562	−0.061	−0.202
**Weekly gel consumption [mL]**	2.0 ± 1.4	2.8 ± 0.5	−9.768	0.002	−1.026	−0.682	−0.761
**Weekly muesli bar [g] consumption**	1.9 ± 1.3	2.8 ± 0.6	−9.964	0.003	−1.051	−0.705	−0.889

Unity means one portion. A serving means one serving of a meal. The frequency with which each food/drink is consumed is indicated in each item. A scale of 1 to 5 was used for the measurement of the variables cook, most days, and satisfaction with weight, with 1 representing the least representative value and 10 representing the maximum.

**Table 3:** Comparative analysis of psychological, body satisfaction, and health variables among groups from a wellness perspective.

					95% Confidence Interval of the Difference		Cohen’s D
Variable	Lower Training Group	Higher Training Group	T	P	Lower	Upper	
**Extraversion [Big Five]**	5.0 ± 1.7	5.6 ± 1.7	−4.239	0.002	−0.877	−0.321	−0.353
**Pleasant [Big Five]**	5.2 ± 2.0	6.3 ± 1.5	−7.934	0.001	−1.457	−0.879	−0.622
**Scrupulous [Big Five]**	6.1 ± 2.1	7.0 ± 1.7	−5.836	0.003	−1.236	−0.613	−0.471
**Neuroticism [Big Five]**	4.9 ± 2.3	5.5 ± 1.9	−3.516	0.004	−0.968	−0.274	−0.284
**Openness to Experience [Big Five]**	6.0 ± 2.2	7.2 ± 1.8	−7.097	0.001	−1.507	−0.853	−0.597
**Zung score**	49.0 ± 5.5	47.0 ± 5.3	4.508	0.000	1.132	2.881	0.370
**AAQII**	21.0 ± 8.7	21.2 ± 9.0	−0.373	0.710	−1.697	1.155	−0.023
**UCLA**	4.2 ± 1.5	4.3 ± 1.6	−0.601	0.548	−0.334	0.177	−0.064
**STAI**	11.8 ± 3.7	11.6 ± 3.5	0.829	0.407	−0.338	0.832	0.056
**Body satisfaction [EDI]**	17.2 ± 3.8	19.0 ± 2.9	−6.614	0.000	−2.391	−1.296	−0.533
**Days you have been injured in the last year**	5.3 ± 22.6	3.3 ± 5.9	1.503	0.133	−0.622	4.677	0.121
**Smoking**	2.3 ± 0.8	2.3 ± 0.8	0.111	0.912	−0.128	0.143	−0.351
**Experience frequent gastritis or heartburn**	1.8 ± 0.7	2.0 ± 0.4	−5.270	0.000	−0.363	−0.166	−0.329
**Frequent dry throat sensation**	1.9 ± 0.7	2.1 ± 0.5	−3.678	0.009	−0.314	−0.095	−0.215
**Frequent dental sensitivity**	1.9 ± 0.7	2.1 ± 0.6	−2.990	0.003	−0.284	−0.058	−0.307
**Days sick throughout the year**	3.6 ± 7.5	2.7 ± 5.7	1.547	0.122	−0.226	1.911	0.135

AAQII [Acceptance and Action Questionnaire II]; UCLA [UCLA Loneliness Scale]; STAI [Spielberger State–Trait Anxiety Inventory]; ZUNG [Zung depression scale].

**Table 4:** Analysis of physical activity variables and their relation to body satisfaction from a wellness perspective.

					95% Confidence Interval of the Difference		
Variable	Lower Training Group	Higher Training Group	T	P	Lower	Upper	Cohen’s D
**Number of training sessions per week**	8.1 ± 26.1	3.9 ± 2.3	2.785	0.006	1.237	7.156	0.227
**Average time in minutes of weekly training**	21.7 ± 19.3	165.4 ± 105.5	−23.121	0.7	−155.884	−131.475	−1.895
**Minutes of weekly aerobic training**	126.7 ± 260.2	5.8 ± 13.1	8.072	0.002	91.447	150.248	0.656
**Bench press PR [kg]**	46.1 ± 106.6	5.8 ± 13.1	6.519	0.001	28.124	52.376	0.531
**Back squat PR [kg]**	44.2 ± 40.7	50.7 ± 43.4	−1.186	0.237	−17.348	4.310	−0.154
**Percentage of week below fifty percent of maximum Load**	38.8 ± 53.4	7.7 ± 11.9	7.286	0.004	−1.467	19.783	0.804
**Percentage of week between fifty and seventy percent of maximum load**	19.6 ± 71.6	10.5 ± 15.5	1.695	0.091	4.627	10.253	0.176
**Percentage of week between seventy and eighty-five percent of maximum load**	13.3 ± 20.6	5.9 ± 11.9	5.196	0.004	4.627	10.253	0.440
**Percentage of training carried out above eighty-five percent**	6.8 ± 13.2	14.5 ± 22.1	3.909	0.002	3.858	11.666	−0.423

## Data Availability

Data are contained within the article.
